# Multicomponent Spiral Wound Membrane Separation Model for CO_2_ Removal from Natural Gas

**DOI:** 10.3390/membranes11090654

**Published:** 2021-08-26

**Authors:** Abdul Aiman Abdul Latif, Kok Keong Lau, Siew Chun Low, Babar Azeem

**Affiliations:** 1 CO_2_ Research Center (CO2RES), Department of Chemical Engineering, Universiti Teknologi PETRONAS, Bandar Seri Iskandar 32610, Perak, Malaysia; abdul_17008993@utp.edu.my; 2 School of Chemical Engineering, Universiti Sains Malaysia, Nibong Tebal 14300, Penang, Malaysia; chsclow@usm.my; 3 Department of Chemical Engineering, The University of Faisalabad, Engineering Wing, Faisalabad 38000, Pakistan; engineerbabar.icet@pu.edu.pk

**Keywords:** spiral wound membrane, modeling, multicomponent, succession stage method, CO_2_

## Abstract

A spiral wound membrane (SWM) is employed to separate acid gases (mainly CO_2_) from natural gas due to its robustness, lower manufacturing cost, and moderate packing density compared to hollow fiber membranes. Various mathematical models are available to describe the separation performance of SWMs under different operating conditions. Nevertheless, most of the mathematical models deal with only binary gas mixtures (CO_2_ and CH_4_) that may lead to an inaccurate assessment of separation performance of multicomponent natural gas mixtures. This work is aimed to develop an SWM separation model for multicomponent natural gas mixtures. The succession stage method is employed to discretize the separation process within the multicomponent SWM module for evaluating the product purity, hydrocarbon loss, stage cut, and permeate acid gas composition. Our results suggest that multicomponent systems tend to generate higher product purity, lower hydrocarbon loss, and augmented permeate acid gas composition compared to the binary system. Furthermore, different multicomponent systems yield varied separation performances depending on the component of the acid gas. The developed multicomponent SWM separation model has the potential to design and optimize the spiral wound membrane system for industrial application.

## 1. Introduction

The demand for natural gas as an energy source has increased exponentially due to its safe, clean, and efficient conversion properties [1,2]. It is widely used for the generation of heat and electricity. The variation in the composition of raw natural gas poses a major challenge for transportation and processing operations. The acid gas in natural gas does not only reduce the calorific value, it imparts acidic properties in the gas (when combined with water [3]) that causes corrosion issues in pipelines and processing equipment. The removal of acid gases from natural gas, therefore, warrants better transportation and processing operations. Table 1 summarizes the typical pipeline specifications for natural gas.

In recent years, gas separation membranes have become a “game changing” technology in the natural gas sweetening process due to their low capital and operating costs [5,6,7,8,9]. The emergence of membrane-based gas separation technology is driven by several factors [5,6] that include: (i) the synthesis of material for high-performance polymer membranes; (ii) large scale production techniques for high-flux asymmetric membranes; and (iii) fabrication techniques for high surface area membrane permeators.

Most commercial membrane modules are manufactured as spiral-wound or hollow fiber configurations due to their high area-to-volume ratio. The manufacturing process of hollow fiber modules is complicated compared to SWM modules. Therefore, only a limited number of materials can be used to fabricate hollow fiber modules. The SWM module is often preferred as it can handle escalated pressure and possesses a higher resistance to fouling that results in a longer life span [7,8]. SWM modules have a long history of CO_2_/CH_4_ separation and are widely commercialized by several manufacturers [6,8,9]. Table 2 summarizes the main current membrane gas separation applications corresponding to the material of the SWM module and its manufacturers.

**Figure 1:**
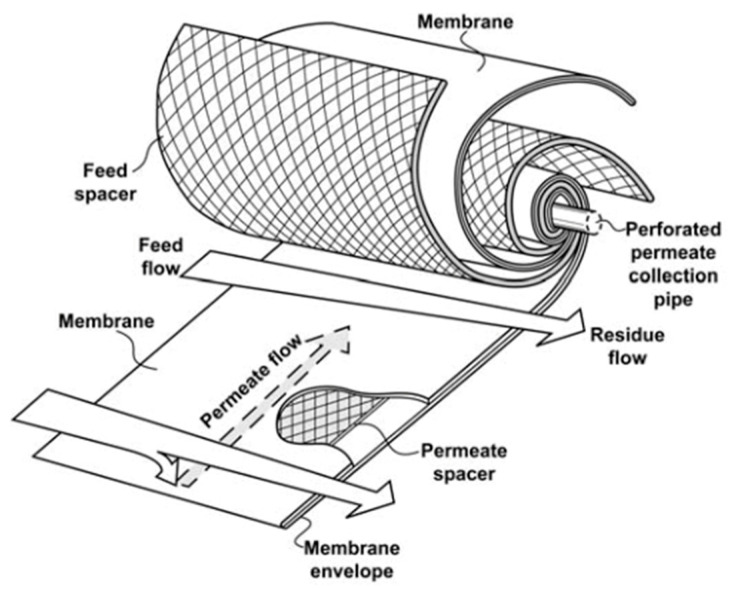
Schematic diagram of a spiral wound membrane [9].

In an SWM module, the membrane is folded around the feed spacer, with the permeate spacer placed in the bottom. Collectively, these layers are known as “leaf” which is later wound around the permeate collection tube for a number of turns as shown in Figure 1. The modules are eventually placed within a cylindrical pressure vessel. Depending on the system arrangement, the modules can be connected either in series or in parallel. 

Typically, high-pressure feed mixture is introduced into the feed channel to facilitate the separation process. The feed spacer provides mechanical support for the membrane sheets and facilitates the high pressure of the retentate stream linearly along the module length. The flow in an SWM module is characterized by a crossflow mechanism, in which the permeation occurs perpendicular to the membrane. As the feed mixture flows axially across the feed spacer, more permeable components permeate through the membrane sheet to the permeate channel. The membrane sheet acts as a selective barrier that separates high-pressure feed mixture and low-pressure permeate. The permeate spacer aids the flow of low-pressure permeate. Subsequently, the permeate flows radially towards the perforated permeate collection tube while the less permeable components retained on the feed side will continue to flow axially and exit as retentate or product at high pressure. 

Lack of widespread precedents for the industrial implementation of membranes in the acid gas sweetening process adds risk to the adaptation of the technology. Therefore, accurate computational models can help to decrease this risk. Despite predicting the separation performance of the module, the adaptation of reliable mathematical models can minimize the technical risks that are inherent in the design of the new process [10]. Additionally, it eliminates need for the time consuming and costly pilot plant and experimental studies [11].

The improved operation of an SWM module requires the derivation of an efficient separation model that can be used to investigate the separation performance of the module. The geometrical arrangement of an SWM module is relatively complex as it involves multiple domains per leaf where variables depend on the relationship of the two-dimensional flow inside spacer channels, making a complete solution intractable. The classic approach is to neglect the curvature of the channels and to consider flow through two flat spacer-filled channels on either side of the membrane. According to Rautenbach et al. [12], this assumption can be justified because the ratio of channel height to the mean module diameter is small. In line with the common industrial practice, constant flow areas are usually assumed.

In gas separation, the available SWM models are based only on binary gas separation. The major development in the methodology used to describe the gas separation process within the SWM module is shown in Table 3. Pan [13] reported a one-dimensional model for gas separation with SWMs that accounts for the permeate pressure drop by means of Darcy’s law. The process variables were found to be dependent only in the x-direction. In another work, Qi and Henson [14] presented an approximate SWM model for binary separation that was developed by the simplification of the simulation model described by Pan [13], while assuming that the flow rate in the feed channel was constant in the spiral direction. Krovvidi et al. [15] developed a model that described a binary gas mixture separation mechanism for both hollow fibers and SWM modules. In terms of operating condition, Safari et al. [16] evaluated the effects of feed temperature, feed pressure, and permeate pressure on the separation performance. Furthermore, more sophisticated models have also been focused on pervaporation applications, where the liquid boundary layer resistance on the retentate side was non-negligible. Lin et al. [17] presented a binary gas separation model that is suitable for SWMs, by using an integral transform from the N–S and mass transfer differential equations. Findings showed that the concentration polarization and structure of SWMs could be neglected. As in the study presented by Gholami et al. [18], mathematical modeling of the binary gas separation process in carbon molecular sieve membranes has been conducted. Results demonstrated that the increase in the effective area, membrane temperature, and total feed pressure would increase the recovery of the fast component in the permeate side, while the feed flow rate had an adverse effect. The study of comprehensive computational fluid dynamics (CFD) was also conducted by Qadir and Ahsan [19] in order to analyze membrane-based gas separation for binary gas mixtures. In the most recent work, Dias et al. [20] presented a simplified approach of a 2D permeation model for SWMs in binary gas separation applications. Pressure variation in both feed and permeate spacer channels was neglected as it was assumed insignificant along a single membrane leaf.

Although membranes are often used to separate multicomponent mixtures, most of the developed SWM models for gas separation have used simplified gas permeation models by assuming binary component separation. In this work, the succession of state approach will be used to establish a two-dimensional mathematical model for multicomponent gas separation using an SWM module to evaluate the product purity, hydrocarbon loss, stage cut, and permeate acid gas composition under varied operating conditions. Furthermore, the separation performance between the binary and multicomponent system will be analyzed and discussed.

## 2. Model Development

### 2.1. Mathematical Modelling

An approximate model was developed to characterize multi-component gas separation for SWM modules in conjunction with the mass balance equation. The succession of states approach was selected due to its simple implementation and lower computational time requirement. This method reduces the problem into finite elements by assuming a constant mass transfer at each element [21]. Each element is independent of one another whereas the outlet condition of the element is computed based on a specific inlet condition that later becomes an inlet condition for the subsequent element. The mass balance and transport equations are computed over every finite element in the matrix to obtain the rate of permeation and composition. The simplicity of this methodology ensures stability and convergence of the algorithm. Furthermore, it enables the non-ideal effects, such as pressure drop and pressure buildup, to be implemented conveniently in conjunction with the mass balance equations.

The solution–diffusion model is used to characterize the transport mechanism of the gas components within the membrane module (see Equation (1)),

(1) $$J_{n}=\frac{P_{n}}{l}\;\left(p_{h}x_{n}-p_{l}y_{n}\right)$$

where $J_{n}$ is the flux of gas component n across the membrane, $P_{n}$ is the permeability of gas component *n*, l is the thickness of the active layer of the membrane, and $p_{l}$ and $p_{h}$ respectively are the low-side and high-side pressures of the membrane module. $x_{n}$ and $\;y_{n}$ are defined as the local retentate and permeate composition of component n at the boundary of the active membrane, respectively.

The following assumptions were made in the proposed model:

○ Local permeation and bulk permeate flow are described by a crossflow pattern [22,23];
○ There is no permeate mixing in the direction of the bulk permeate flow [19,23];
○ The pressure variation for flow through the permeate spacer channel is characterized by Hagen–Poiseuille equation [24];
○ The feed-side pressure drop is negligible [15,16,19,20,23,24];
○ The pressure drop along the central permeate collector tube is neglected (i.e., pressure is considered as atmospheric) [25,26];
○ The permeability coefficients are independent of concentration, pressure, and temperature [15,16,19,20,23,24];
○ Channel curvature is neglected and the membrane is treated as a flat sheet [22,26];
○ Operation within the SWM module is isothermal [16,18,19,20,27].

To compute the active membrane area ($A_{m}$) based on a predetermined number of elements, the membrane dimension specifications, as below, are fixed prior to calculation.

1. Channel spacer, $h_{p},\;h_{f}$ and membrane, δ thickness;
2. Module, $d_{o}$, and collection tube, $d_{i}$ diameter;
3. Number of leaves, k;
4. Module length, L;

The active membrane area of each element, as computed using Equations (2)–(5), depends upon the number of leaves and dimensions of the membrane itself.

(2) $$Thickness\;of\;envelopes,h=kh_{p}+2k\delta +kh_{f}\;\;$$

(3) $$Number\;of\;turn,N=\left(\frac{d_{o}-d_{i}}{2h}\right)+1$$

(4) $$Membrane\;width,W=\pi \left[Nd_{i}+hN\left(N-1\right)\right]$$

(5) $$Active\;membrane\;area,A_{m}=L\times W$$

**Figure 2:**
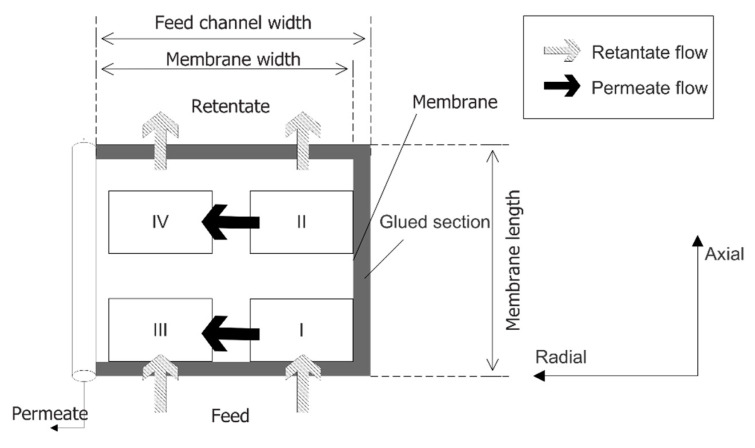
Succession of state approach in an SWM.

The succession of state methodology divides the SWM module into four types of elements as shown in Figure 2. The mass computation for each type is different. Type I is referred to as the first element that interacts with the feed, located at the corner of membrane sheet (near the glued side). Type II is located along the glued side’s axial direction, while Type III is situated along the entrance feed on the radial direction. Type IV represents remaining elements in the radial and axial directions in the membrane module. The computation procedures are started at the entrance elements (Type I and Type III, where the initial concentration along the membrane width is identical) to compute the mass transfer across the membrane for the first element before proceeding to the next element. It ends as it covers the entire membrane module.

**Figure 3:**
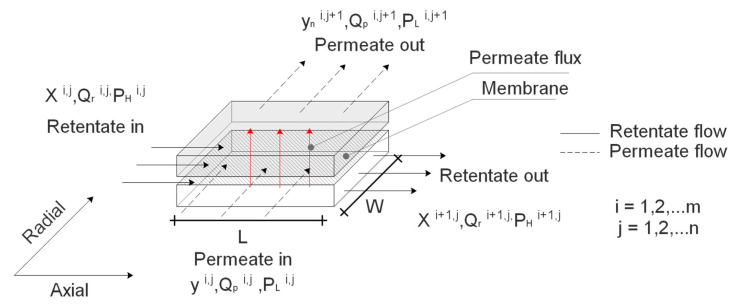
Schematic representation of an elemental volume in the cross-flow SWM element.

Figure 3 represents the elemental volume of the cross-flow SWM module elements utilized in the proposed model that is comprised of the feed and permeate sections and membrane layer. The axial direction is represented by the dimension i while the radial direction is represented by the dimension j. The leaf is represented by the dimension k. The components are differentiated in terms of their permeating nature. The fast-permeable component is referred to as x in the retentate stream, while y is the permeable component in the permeate stream.

The characterization of transport across the cross-flow SWM module element using Fick’s law of diffusion, is described in Equation (6):

(6) $$y_{n}\left(i,j,k\right)=\;\frac{P_{n}A_{m}\left(\frac{p_{h}}{1-\theta^{*}}\left(x_{n}\left(i,j-1,k\right)-\theta^{*}y_{n}\left(i,j,k\right)\right)-p_{l}y_{n}\left(i,j,k\right)\right)}{Q_{r}\left(i,j-1,k\right)\theta^{*}}$$

Coupled with the Newton bisection numerical solution, the guessed permeate compositions $y_{1}\left(i,j,k\right)$ are initiated from the low and high sides to ensure quick convergence. 

For Type I and Type II cells at various axial positions along the closed-end membrane sheet, the permeate composition of each component, $y_{n}\left(i,j,k\right)$, is determined using the solution procedure outlined. After determining permeate composition, total flow rate of each component into the permeate stream, ${\dot{Q}}_{n}\left(i,j,k\right)$, the flow rate into the permeate stream, ${\dot{Q}}_{p}\left(i,j,k\right)$, the retentate stream flow rate, ${\dot{Q}}_{r}\left(i,j,k\right)$, and the retentate stream composition, $x_{n}\left(i,j,k\right)$, are estimated using Equations (7)–(11):

(7) $${\dot{Q}}_{n}\left(i,j,k\right)=P_{n}A_{m}\left(p_{h}x_{n}\left(i-1,j,k\right)-p_{l}\left(i,j,k\right)y_{n}\left(i,j,k\right)\;\right)$$

(8) $$\Delta \dot{Q\left(i,j,k\right)}=\sum_{n=1}^{n}P_{n}A_{m}\left(p_{h}x_{n}\left(i-1,j,k\right)-p_{l}\left(i,j,k\right)y_{n}\left(i,j,k\right)\;\right)$$

(9) $${\dot{Q}}_{p}\left(i,j,k\right)=\Delta \dot{Q}\left(i,j,k\right)$$

(10) $${\dot{Q}}_{r}\left(i,j,k\right)={\dot{Q}}_{r}\left(i-1,j,k\right)-{\dot{Q}}_{p}\left(i,j,k\right)$$

(11) $$x_{n}\left(i,j,k\right)=\frac{{\dot{Q}}_{r}\left(i,j,k\right)x_{n}\left(i,j-1,k\right)-{\dot{Q}}_{p}\left(i,j,k\right)}{{\dot{Q}}_{r}\left(i,j,k\right)}$$

For Type I cells in direct contact with the feed side, the indices $x_{n}\left(i-1,j,k\right)$ and ${\dot{Q}}_{r}\left(i-1,j,k\right)$ are replaced with the feed condition.

Similarly, for Type III and IV cells, the permeate composition of each component in a gaseous mixture, $y_{n}\left(i,j,k\right)$, is determined using the algorithm proposed. Later, the total flow rate of each component into the permeate stream, ${\dot{Q}}_{n}\left(i,j,k\right)$, the flow rate into the permeate stream, ${\dot{Q}}_{p}\left(i,j,k\right)$, the retentate stream flow rate, ${\dot{Q}}_{r}\left(i,j,k\right)$, and the retentate stream composition, $x_{n}\left(i,j,k\right)$, contacting the subsequent cells are calculated using Equations (12)–(16):

(12) $${\dot{Q}}_{n}=P_{n}A_{m}\left(p_{h}x_{n}\left(i-1,j,k\right)-p_{l}\left(i,j,k\right)y_{n}\left(i,j,k\right)\;\right)$$

(13) $$\Delta \dot{Q}=\sum_{n=1}^{n}P_{n}A_{m}\left(p_{h}x_{n}\left(i-1,j,k\right)-p_{l}\left(i,j,k\right)y_{n}\left(i,j,k\right)\;\right)$$

(14) $${\dot{Q}}_{p}\left(i,j,k\right)=\Delta \dot{Q}\left(i,j,k\right)+{\dot{Q}}_{p}\left(i,j-1,k\right)$$

(15) $${\dot{Q}}_{r}\left(i,j,k\right)={\dot{Q}}_{r}\left(i-1,1,k\right)-{\dot{Q}}_{p}\left(i,j,k\right)$$

(16) $$x_{n}\left(i,j,k\right)=\frac{{\dot{Q}}_{r}\left(i,j,k\right)x_{n}\left(i,j-1,k\right)-{\dot{Q}}_{p}\left(i,j,k\right)}{{\dot{Q}}_{r}\left(i,j,k\right)}$$

For the Type III cells in direct contact with the feed, the indices $x_{n}\left(i-1,j,k\right)$ and $x_{n}\left(i-1,j,k\right)$ are replaced with the feed condition.

Besides, the permeate spacer channel pressure variation is characterized by the Hagen–Poiseuille equation, as presented in Equation (17):

(17) $$\Delta p_{l}\left(i,j,k\right)=\;\lambda \frac{v^{2}\rho L}{2d_{h}}$$

Where λ is the friction coefficient, ρ is the specific density, v is the linear velocity, L is the length of membrane, and $d_{h}$ is the hydraulic diameter. The friction coefficient is a function of Reynolds number as presented in Equation (18):

(18) $$\lambda =44Re^{-0.55}$$

The classical Reynolds number (*Re*) expresses the relationship between turbulence flow and fluid viscosity:

(19) $$Re=\frac{\rho \nu d_{h}}{\mu }$$

The hydraulic diameter ($d_{h}$) for spacer filled channels on the feed and permeate sides are calculated using Equation (20):

(20) $$d_{h}=\frac{4\cdot \emptyset }{\frac{2}{h_{ch}}+\frac{1-\emptyset }{8\cdot h_{ch}}}$$

The linear flow velocity (v) is computed as follows:

(21) $$v=\frac{Q}{A}\;=\frac{Q}{w_{ch}h_{ch}}$$

where v is the linear flow velocity, Q is the feed flow rate, and A is area of the feed channel cross section, which is the product of the channel width $\left(w_{ch}\right)$, height $\left(h_{ch}\right)$, and porosity (∅) of the flow channel.

The viscosity of gas mixture is calculated by adapting Wilke’s method [28,29], while the viscosity of the pure components is determined using Lucas’s method [30,31].

### 2.2. Simulation Method

In this work, the proposed model is developed using MATLAB R2020a for the simulation of the separation process within the multicomponent SWM module for evaluating the product purity, hydrocarbon loss, stage cut, and permeate acid gas composition. The model is validated using experimental data from Baker [32]. Three simulation cases are established: binary case; multicomponent—Case 1 (4 components), and multicomponent—Case 2 (5 components). The parametric analysis of the influence of the operating conditions (feed pressure, feed flow rate, and acid gas composition) on the separation performance for given characteristics of an SWM module, are reported. Unless specified, the simulations are run with the input parameters as summarized in Table 4. 

The operating condition of natural gas processing depends mainly on the source. The characteristics of raw natural gas depend solely on the origin, location of deposit, and geological structure. Ranging from 28 mol% to 87 mol% of CO_2_ contents, 13 trillion cubic feet of natural gas reserves are reported undeveloped in Malaysia [33]. Hence, the feed composition for a natural gas stream is varied from 10 mol% to 70 mol% in the current work. The feed flow rate for a single SWM module is varied from 0.2 MMSCFD to 2 MMSCFD. A typical feed pressure (15–60 bar) for the membrane separation system is used in the simulation.

## 3. Results and Discussion

### 3.1. Element Sensitivity Analysis

During the computation of the model, the current succession of state approach assumed the subdivision of a large system into smaller elements with the dependence of mass across a single element on the inlet condition of that particular cell. As numerical solutions obtained through discretization methods can accumulate truncation, rounding, and inherited errors, element sensitivity analysis is necessary to evaluate which numerical configuration (number of discretized elements along the radial and axial directions) leads to an accurate model. 

**Figure 4:**
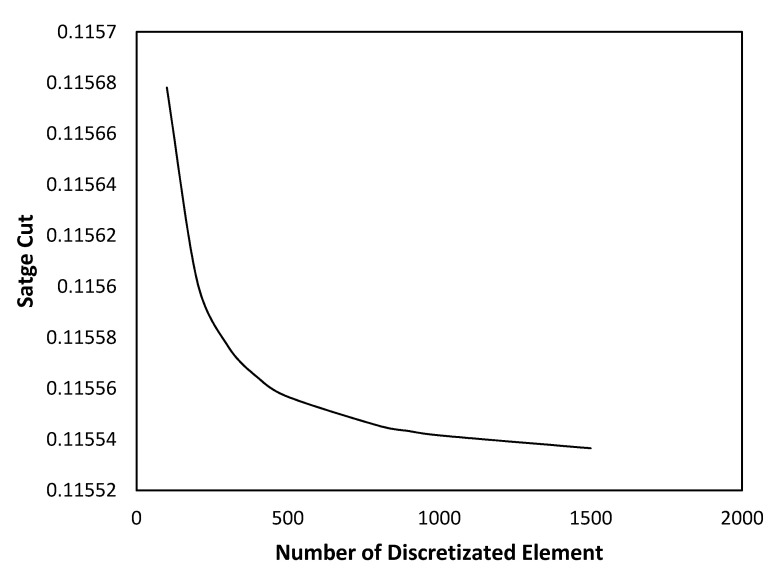
Element sensitivity analysis.

Figure 4 shows the element sensitivity analysis. An increasing number of discretized elements along the radial and axial direction would reduce the size of the individual elements. It is observed that for 1000 elements (1000 × 1000 elements) and above, the value for the simulated stage cut remains relatively constant, showing the simulation error is within the acceptable limit. Based on this result, the number of elements along the radial and axial directions are set at 1000 (1,000,000 elements overall) for the current parametric study.

### 3.2. Model Validation

Table 5 shows a comparison between the experimental and modeled values for gas separation process using an SWM. In the experimental work [32], a total active membrane area of 330 cm^2^ polyamide copolymer membrane was incorporated into a bi-module spiral wound membrane (connected in series) and fed with different flow rates (51,500 cm^3^/min, 18,500 cm^3^/min, and 10,100 cm^3^/min). The feed compositions for the modules consisted of 25 mol% hydrogen (H_2_) and 75 mol% carbon dioxide (CO_2_) at 25 °C. The feed pressure was maintained at 100 psig.

The simulated retentate compositions of carbon dioxide (CO_2_) and hydrogen (H_2_) were compared with the corresponding experimental values at varying feed flow rates. The simulated results demonstrated good agreement with the experimental data with a percentage error range from 0.4472% to 2.6099% for CO_2_ and 1.2595% to 6.2373% for H_2_, respectively. The maximum average percentage error (MAPE) for both components are less than 3%. These MAPE values are considered acceptable compared to other similar numerical modeling works [20,21,22], which confirms the validity of the numerical model to be employed in the subsequent parametric analysis. 

#### 3.2.1. Effect of Feed Pressure

**Figure 5:**
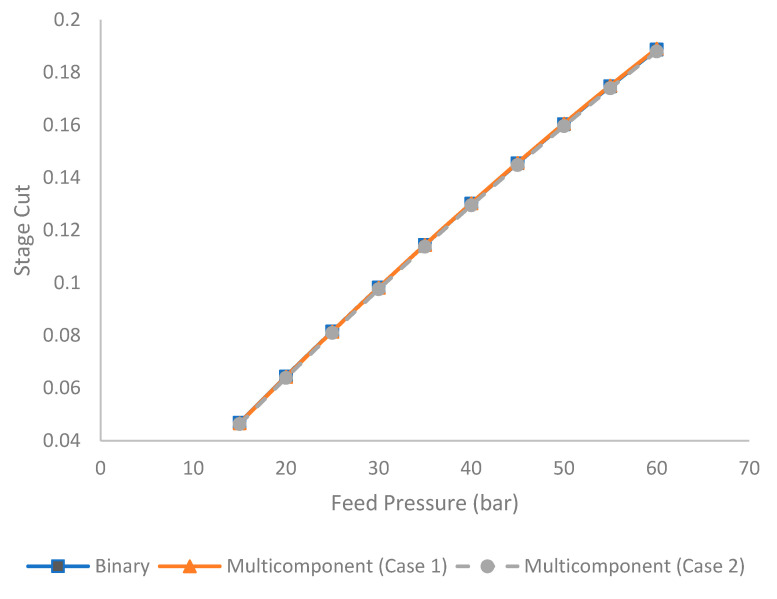
Evolution of stage cut under different feed pressures for binary and multicomponent systems.

Figure 5 shows the evolution of stage cut under different feed pressures for binary and multicomponent systems (Case 1 and 2). Stage cut represents a ratio between permeate flow rate and feed flow rate presented as follows:

(22) $$\mathrm{Stage}\;\mathrm{cut}=\frac{\mathrm{Permeate}\;\mathrm{flow}\;\mathrm{rate}}{\mathrm{Feed}\;\mathrm{flow}\;\mathrm{rate}}$$

It is observed in Figure 5 that an increase in feed pressure tended to enhance the stage cut, which is contributed by the higher driving force for the gas permeation. Based on the solution diffusion model, the increase in feed pressure enhances the partial pressure that subsequently generates a higher chemical potential for gas permeation [29,41]. The stage cut difference between binary and multicomponent (Case 1 and 2) is not significant because the total amount of acid gas in binary and multicomponent systems (Case 1 and 2) is identical.

**Figure 6:**
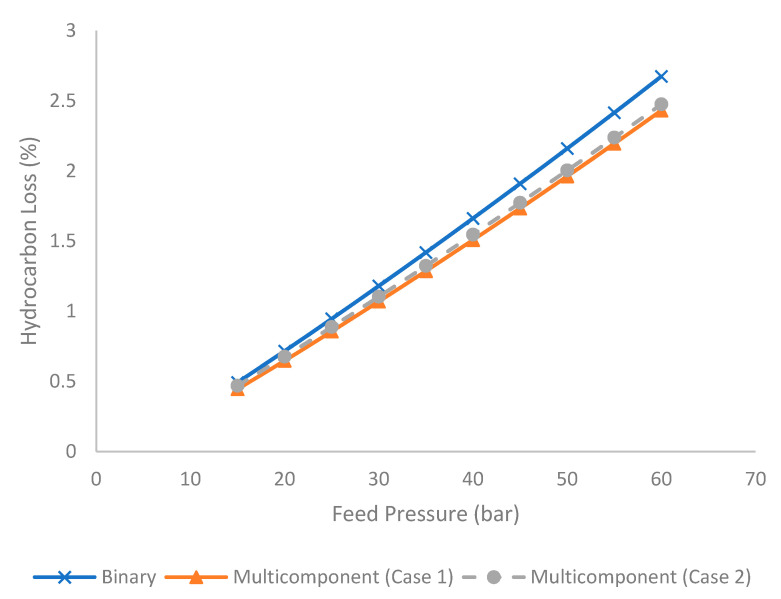
Evolution of hydrocarbon loss (%) under different feed pressures for binary and multicomponent systems.

Figure 6 shows the evolution of hydrocarbon loss under different feed pressures for binary and multicomponent systems (for Case 1 and 2). For the acid gas sweetening process, it is desirable to have a low hydrocarbon loss to maximize hydrocarbon recovery. Hydrocarbon loss is computed using Equation (23):

(23) $$\mathrm{Hydrocarbon}\;\mathrm{Loss}\;\left(\%\right)=\frac{\mathrm{Hydrocarbon}\;\mathrm{in}\;\mathrm{the}\;\mathrm{permeate}}{\mathrm{Hydrocarbon}\;\mathrm{in}\;\mathrm{the}\;\mathrm{feed}}\%$$

It is observed in Figure 6 that an increase in feed pressure contributed to higher hydrocarbon loss. This observation is in line with the evolution of stage cut, in which higher stage cut (due to higher feed pressure) tended to generate higher hydrocarbon loss under constant selectivity [16]. In addition, the binary system generated higher hydrocarbon loss compared to the multicomponent system. This is due to the presence of a higher composition of methane as the most permeable hydrocarbon component in the binary mixture, compared to a multicomponent system. Under current analysis, the binary mixture contains 60 mol% of methane, whereas the multicomponent system consists of 50 mol% of methane and 10 mol% of ethane and propane. 

**Figure 7:**
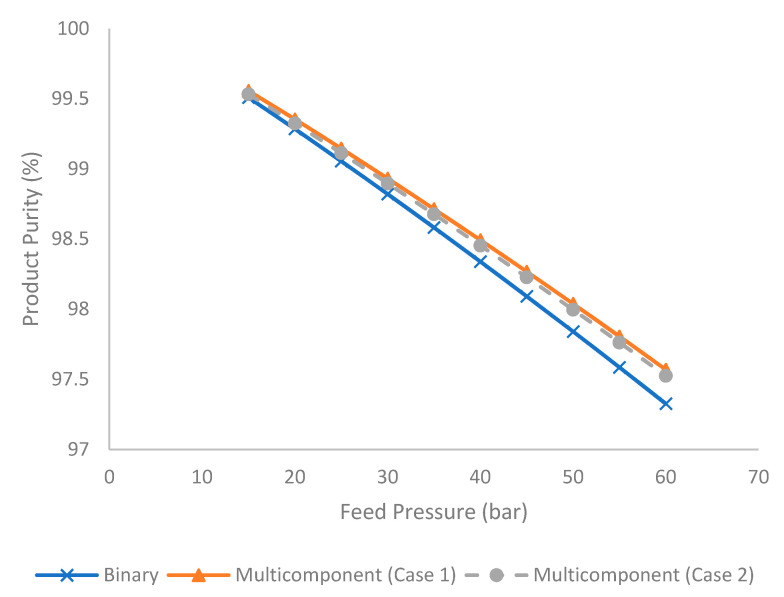
Evolution of product purity (%) under different feed pressure for binary and multicomponent systems.

Figure 7 illustrates the evolution of product purity under different feed pressures for binary and multicomponent systems (Case 1 and 2). Product purity is estimated using Equation (24):

(24) $$\mathrm{Product}\;\mathrm{Purity}\;\left(\%\right)=\frac{\mathrm{Hydrocarbon}\;\mathrm{in}\;\mathrm{product}\;\mathrm{stream}}{\mathrm{Total}\;\mathrm{product}\;\mathrm{stream}}\%$$

It is observed in Figure 7 that an increase in feed pressure tended to reduce the product purity. This was contributed to by the increase in stage cut and hydrocarbon loss that reduced the hydrocarbon content in the product stream. Furthermore, the binary system tended to produce lower product purity compared to the multicomponent system. This was due to the presence of a higher composition of methane (as the most permeable hydrocarbon component) in the binary mixture as explained earlier.

**Figure 8:**
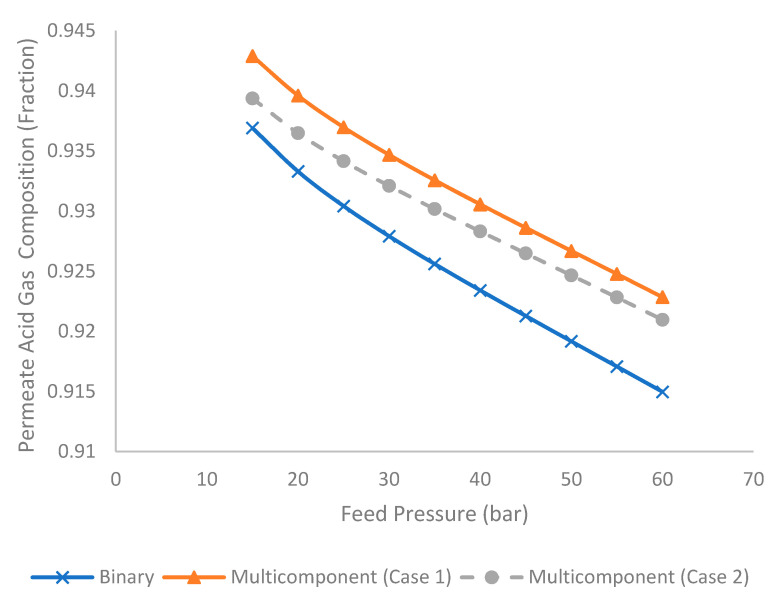
Evolution of permeate acid gas composition under different feed pressure for binary and multicomponent system.

Figure 8 shows the permeate acid gas composition at different feed pressures for binary and multicomponent systems (Case 1 and 2). Typically, higher permeate acid gas compositions were required to fulfil the cold venting or sequestration requirement. Figure 8 indicates that the increase in feed pressure reduced the permeate acid gas composition. This was contributed to by an increase in hydrocarbon loss to the permeate stream under the higher stage cut that diluted the concentration of acid gas in the permeate stream. The binary system, compared to different gas mixture systems, tended to generate a lower acid composition because it possessed a higher composition of methane compared to a multicomponent system. A comparison of the multicomponent systems revealed that Case 1 tended to produce a higher permeate acid gas composition because it consisted of a higher composition of CO_2_ (a component that is more permeable than H_2_S [34]). In the current study, multicomponent Case 1 contains 40 mol% of CO_2_, whereas the multicomponent Case 2 consists of 30 mol% of CO_2_ and 10 mol% of H_2_S. 

#### 3.2.2. Effect of the Feed Flow Rate

**Figure 9:**
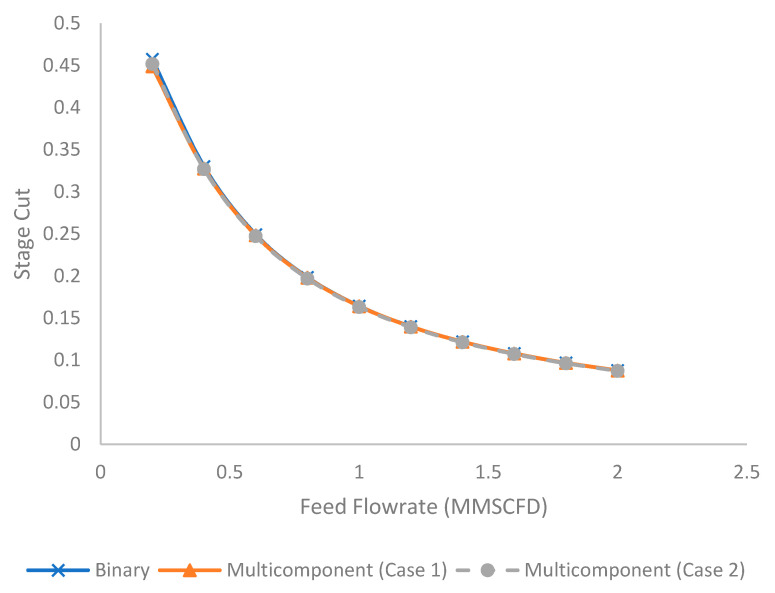
Evolution of stage cut under different feed flow rates for binary and multicomponent systems.

Figure 9 illustrates the evolution of stage cut under the different feed flow rates for binary and multicomponent systems (Case 1 and 2). Since the stage cut is defined as the ratio between the permeate flow and feed flow rate (Equation (22)), increase in feed flow rate reduces the stage cut assuming a constant chemical potential across the membrane [15,42,43]. In addition, since the total amount of acid gas in binary and multicomponent systems (Case 1 and 2) is identical, the stage cut difference between binary and multicomponent (Case 1 and 2) is not significant. 

**Figure 10:**
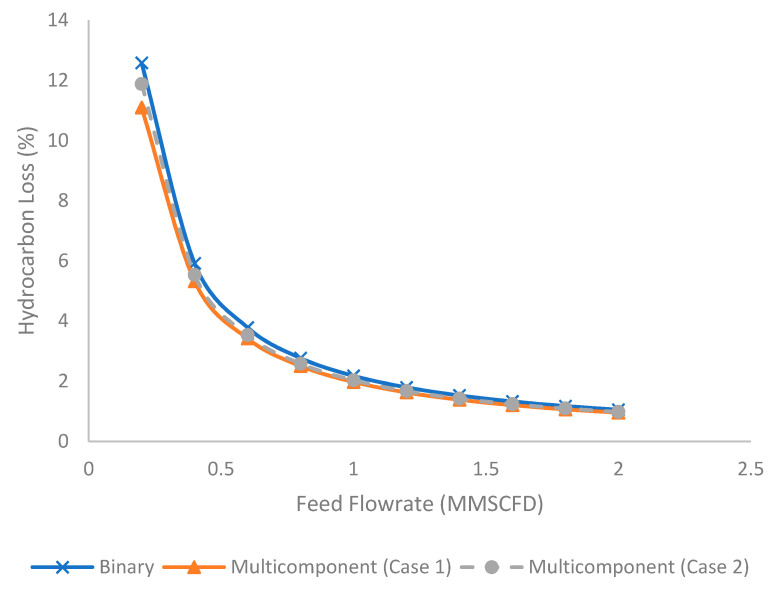
Evolution of hydrocarbon loss (%) under different feed flow rates for binary and multicomponent systems.

Figure 10 represents the evolution of hydrocarbon loss (%) at different feed flow rates for binary and multicomponent systems. The increase in feed flow rate tended to reduce the hydrocarbon loss. The reduction in stage cut at the higher feed flow rate produced lower permeation that reduced hydrocarbon loss. The binary system produced higher hydrocarbon loss compared to the multicomponent system due to the presence of a higher composition of methane (the most permeable hydrocarbon component). The differences became less significant when the flow rate increased, which was attributed to the reduction in stage cut.

**Figure 11:**
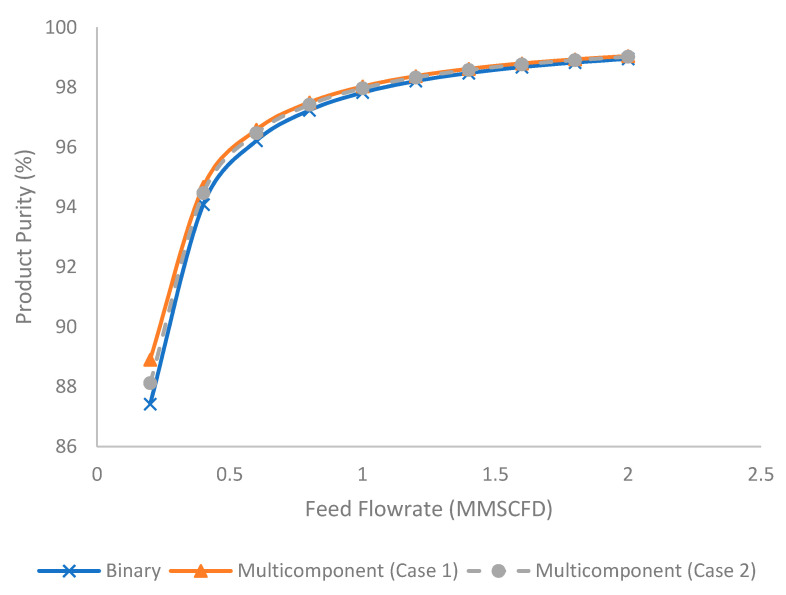
Evolution of product purity (%) under different feed flow rates for binary and multicomponent systems.

Figure 11 illustrates the evolution of product purity (%) at different feed flow rates for binary and multicomponent systems. The increase in feed flow rate increased the product purity. This was due to the reduction in stage cut and hydrocarbon loss that increased the hydrocarbon content in the product stream. The binary system tended to produce lower product purity compared to the multicomponent system due to the presence of a higher composition of methane as explained earlier.

**Figure 12:**
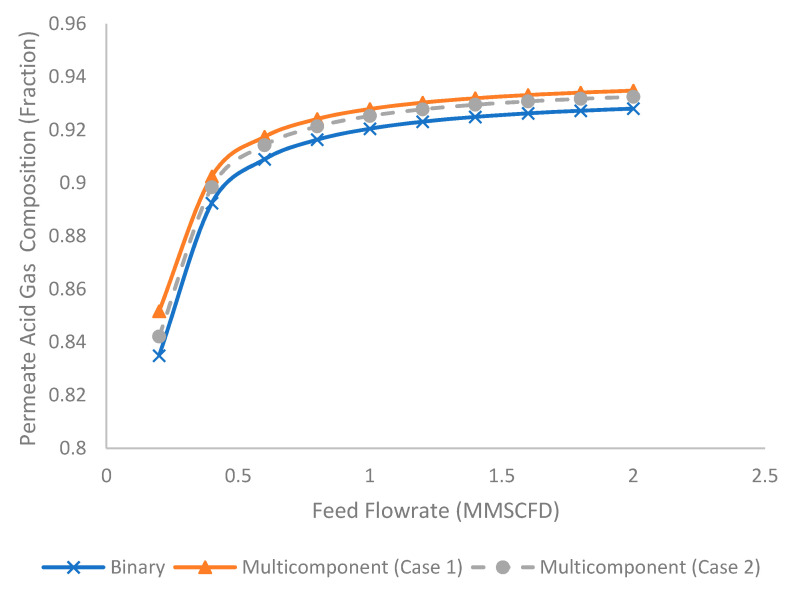
Evolution of permeate acid gas composition under different feed flow rates for binary and multicomponent systems.

Figure 12 shows the permeate acid gas composition under different feed flow rates for binary and multicomponent systems. The increase in feed flow rate increased the permeate acid gas composition. This was due to the reduction in hydrocarbon loss to the permeate stream under lower stage cut that increased the concentration of acid gas in the permeate stream. The binary system tended to generate the lowest acid gas composition, followed by multicomponent Case 2 and Case 1. This was due to the presence of a higher composition of methane in the binary system and lower permeating acid gas (H_2_S) in the multicomponent system, Case 2, as explained in the earlier section.

#### 3.2.3. Effect of Acid Gas Composition

**Figure 13:**
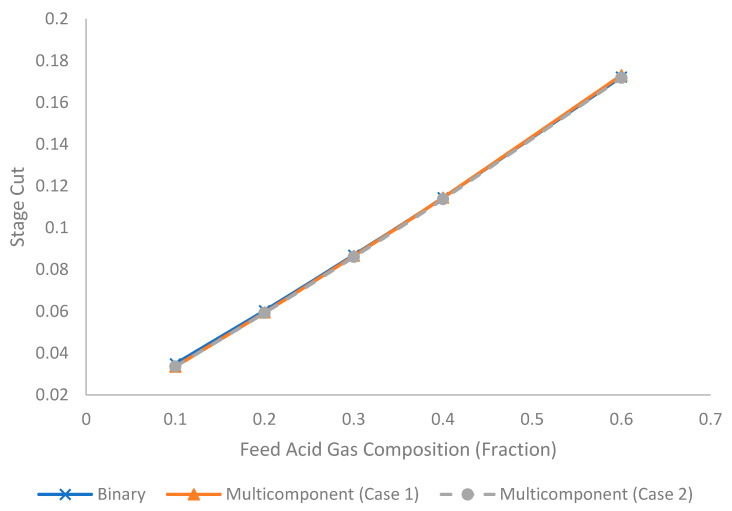
Evolution of stage cut under different acid gas composition (fraction) for binary and multicomponent systems.

Figure 13 illustrates the evolution of stage cut under different feed acid gas compositions for binary and multicomponent systems (Case 1 and 2). CO_2_ is the only acid gas component in the binary system and Case 1. For Case 2, the acid gases consisted of CO_2_ and H_2_S with a molar ratio of 3:1. Since the acid gases are more permeable than hydrocarbons, the increase in feed acid gas composition tended to increase the stage cut as shown Figure 13. Furthermore, since the total amount of acid gas in the binary and multicomponent systems was identical, the stage cut difference between binary and multicomponent is not significant.

**Figure 14:**
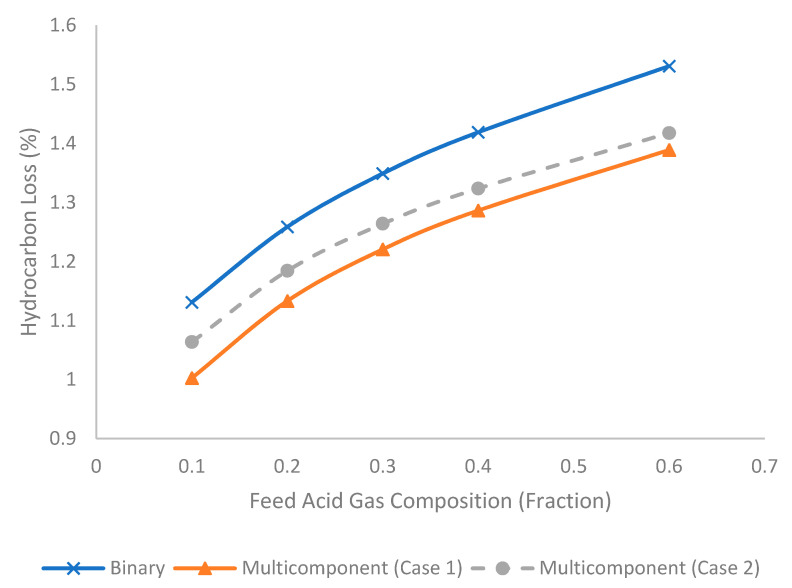
Evolution of hydrocarbon loss (%) under different acid gas composition (fraction) for binary and multicomponent systems.

Figure 14 represents the evolution of hydrocarbon loss (%) under different feed acid gas compositions for binary and multicomponent systems. The increase in feed acid gas composition increased the hydrocarbon loss. As demonstrated earlier, a higher feed acid gas composition increased the stage cut that enhanced the permeation and increased the hydrocarbon loss under a constant selectivity. The binary system produced the highest hydrocarbon loss, followed by multicomponent Case 2 and Case 1, as explained earlier. Based on Equation (17) (Hagen–Poiseuille Equation), the presence of H_2_S in Case 2 lowered the total density of the permeate, which contributed to lower pressure buildup in the permeate stream. This phenomenon generated a higher driving force (or pressure difference between the feed and permeate stream) across the membrane interface, which caused a higher permeation flux. This slight increase in permeation flux contributed to a higher hydrocarbon loss for Case 2 compared to Case 1. 

**Figure 15:**
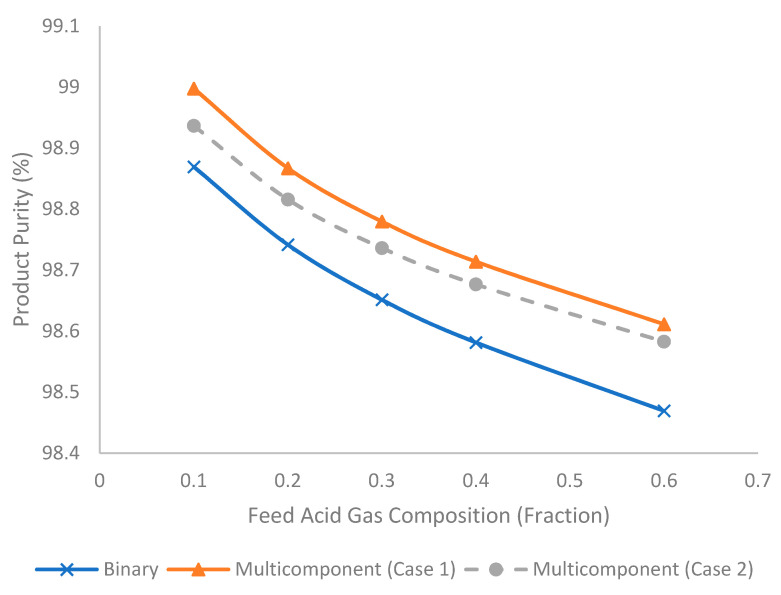
Evolution of product purity (%) under different acid gas composition for binary and multicomponent systems.

Figure 15 illustrates the evolution of product purity under different feed acid gas compositions for binary and multicomponent systems. The increase in feed acid gas composition tended to reduce the product purity due to the increase in stage cut and hydrocarbon loss that reduced the hydrocarbon content in the product stream. In addition, the binary system tended to produce the lowest product purity followed by multicomponent Case 2 and Case 1. This trend is attributed to the presence of a higher composition of methane in the binary system and lower permeating acid gas (H_2_S) in multicomponent Case 2.

**Figure 16:**
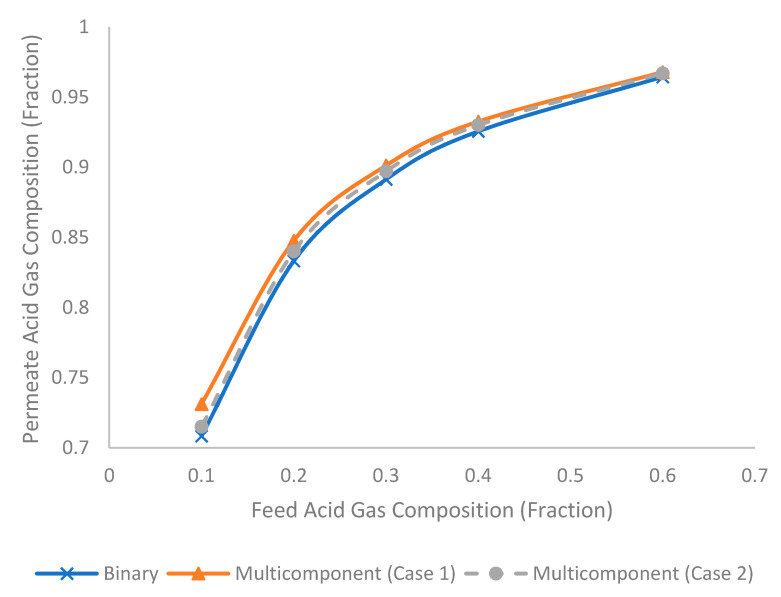
Evolution of permeate acid gas composition (fraction) under different feed acid gas composition (fraction) for binary and multicomponent systems.

Figure 16 shows the permeate acid gas composition under different feed acid gas compositions for binary and multicomponent systems. The increase in feed acid gas composition tended to increase the permeate acid gas composition. This is because the higher feed acid gas composition increased the chemical potential of acid gas to permeate through the membrane, that subsequently increased the permeate acid gas composition [44]. Similar to the earlier discussion, the binary system tended to generate the lowest permeate acid gas composition, followed by multicomponent Case 2 and Case 1.

## 4. Conclusions

A validated spiral-wound-membrane model was developed to describe the separation performance of multicomponent natural gas mixtures. Based on our results, the increase in feed pressure and feed acid gas composition tended to increase the stage cut and hydrocarbon loss. To the contrary, higher feed flow rate reduced the stage cut and hydrocarbon loss. Higher product purity can be achieved from lower feed pressure and feed acid gas composition. Analyzing the separation performance of binary and multicomponent systems, it is observed that multicomponent systems tended to produce higher product purity, lower hydrocarbon loss, and higher permeate acid gas composition compared to the binary system. This is due to the presence of a higher composition of methane (as the most permeable hydrocarbon component) in the binary mixture. A comparison between the multicomponent systems suggests that Case 1 produced a higher permeate acid gas composition because it consisted of a higher composition of CO_2_ that was more permeable than H_2_S. As a way forward, the developed model will be extended to investigate the effect of different module configurations for designing and optimizing the spiral wound membrane system.

## Figures and Tables

**Table 1:** Specifications for pipeline quality natural gas [3,4].

Components	Specifications
Methane	75%
Ethane	10%
Propane	5%
Butanes	2%
Pentane and heavier	0.50%
Nitrogen and other inert	3%
Carbon dioxide	2–3%
Total diluent gas	4–5%
Hydrogen sulfide	6–7 mg/m^3^
Total sulfur	115–660 mg/m^3^
Water vapor	60–110 mg/m^3^
Oxygen	1%

**Table 2:** SWM gas separation applications corresponding to materials and manufacturers [9].

Gas Separation	Application	Membrane Material	Manufacturer
CO_2_/Hydrocarbon	Acid gas treatment, enhanced oil recovery, landfill gas upgrading	Cellulose acetate, polyimide	GMS (Kvaerner), Separex (UOP), Cynara (Natco)
VOC/N_2_	Vapor/gas separation, air dehydration, other	Silicone rubber	Aquilo, Parker-Hannifin, Ube, GKSS Licensees, MTR

**Table 3:** Summary of major developments in methodology adapted to characterize the gas separation performance of SWM modules.

Numerical Work	Study Domain	Assumptions/Limitations
Pan [13]	Perpendicular 1D mass balance between the feed and permeate channels with the consideration of pressure variation in the permeate stream.	- Binary system; 1D mathematical models, which are unsuitable for module optimization due to the requirement of many approximations and assumptions.
Krovvidi et al. [15]	Simplified mass balance model assuming a relationship between the feed and permeate stream concentration.	- Binary system; pressure builds up and is neglected along the permeate stream.
Qi and Henson [14]	1D mass balance simplified by assuming the flow rate in the feed channel is constant in the spiral direction	- Binary system; requires detailed characteristics of the membrane which are often not known at the preliminary design stage.
Safari et al. [16]	Derived simple models for permeability and selectivity variations in the CO_2_/CH_4_ system that include both temperature and pressure effects.	- Binary system.
Lin et al. [17]	Mathematical model for a polydimethylsiloxane spiral wound membrane by using an integral transform from Navier–Stokes and the mass transfer differential equation.	- Binary system; accuracy of the method is only in good agreement with polydimethylsiloxane spiral wound membranes.
Gholami et al. [18]	Modeling of the gas separation process with a flat carbon membrane.	- Binary system.
Qadir and Ahsan [19]	Computational fluid dynamics (CFD) model describes the flow profiles of gases in different membrane modules.	- Binary system; due to the complexity of the model, it requires the highest computational resources for the model solution.
Dias et al. [20]	A 2D mathematical model describes the operation of spiral wound membranes in industrial gas separation processes.	- Binary system; pressure builds up and is neglected along the permeate stream.

**Table 4:** Input parameters used for the simulation of membrane case studies.

Simulation Parameter		Value
Membrane characteristic [34,35]	Permeance of CO_2_ (GPU)	90
	Permeance of CH_4_ (GPU)	4.5
	Permeance of C_2_H_6_ (GPU)	1.8
	Permeance of C_3_H_8_ (GPU)	1.8
	Permeance of H_2_S (GPU)	87.3
Feed gas characteristic	Composition: binary case (mol fraction)	Z_F_; CO_2_ = 0.4
		Z_F_; CH_4_ = 0.6
	Composition: multicomponent Case 1 (mol fraction)	Z_F_; CO_2_ = 0.4
		Z_F_; CH_4_ = 0.5
		Z_F_; C_2_H_6_ = 0.08
		Z_F_; C_3_H_8_ = 0.02
	Composition: multicomponent Case 2 (mol fraction)	Z_F_; CO_2_ = 0.3
		Z_F_; CH_4_ = 0.5
		Z_F_; C_2_H_6_ = 0.08
		Z_F_; C_3_H_8_ = 0.02
		Z_F_; H_2_S = 0.1
	Temperature (C) [36]	40
	Pressure (bar) [36]	35
Output gas characteristic [36]	Permeate pressure (bar)	1.05
Membrane module Characteristic [37,38,39,40]	Feed spacer channel thickness (cm)	9.00 × 10^−2^
	Permeate spacer channel thickness (cm)	4.00 × 10^−2^
	Spacer channel porosity	0.846 (feed channel)
		0.616 (permeate channel)
	Number of envelopes	30
	Module diameter (cm)	20.32
	Module length (m)	1

**Table 5:** Comparison between experimental [32] and modeled values for gas separation using spiral wound membranes.

Operating Conditions		Retentate Composition							
		Carbon Dioxide (CO_2_)				Hydrogen (H_2_)			
Feed pressure (psig)	Feed flow rate (cm^3^/min)	Exp.	Model	Error (%)	MAPE (%)	Exp.	Model	Error (%)	MAPE (%)
100	51,500	0.7380	0.7413	0.4472	1.2268	0.2620	0.2587	1.2595	2.9613
100	18,500	0.7050	0.7234	2.6099		0.2950	0.2766	6.2373	
100	10,100	0.6900	0.6943	0.6232		0.3100	0.3057	1.3871	

## Data Availability

The data presented in this study are available on request from the corresponding author.

## Abbreviations

$A_{m}$	Effective membrane area (m)
$d_{o},d_{i}$	External and internal diameter
$d_{h}$	Wetted diameter
h	Thickness of envelopes
$J_{n}$	Permeation flux
l	Active layer thickness of the membrane
L	Module length
$h_{ch}$	Height channel per element
N	Number of turn(s)
$P_{n}$	Permeability
$p_{h}$	High pressure on the feed side
$p_{l}$	Low pressure on the permeate side
$P_{n}$	Mix gas permeance of each component in the feed
${\dot{Q}}_{f}$	Feed flow rate (m^3^/s)
${\dot{Q}}_{r}\left(i,j,k\right)$	Feed side flow rate cell (m^3^/s)
${\dot{Q}}_{p}\left(i,j,k\right)$	Permeate side flow rate cell
W	Membrane width
$w_{ch}$	Width channel per element
Sum	Summation of all components composition in the permeate side
$x_{n}$	Retentate side composition cell
$y_{n}$	Permeate side composition cell

## References

[1] Hu W., Bao J., Hu B. (2013). Trend and progress in global oil and gas exploration. Pet. Explor. Dev..

[2] Liang F.-Y., Ryvak M., Sayeed S., Zhao N. (2012). The role of natural gas as a primary fuel in the near future, including comparisons of acquisition, transmission and waste handling costs of as with competitive alternatives. Chem. Central J..

[3] Mokhatab S., Poe W.A., Mokhatab S., Poe W.A., Speight J.G. (2006). Chapter 3—Raw gas transmission. Handbook of Natural Gas Transmission and Processing.

[4] Kidnay A.J., Parrish W.R. (2006). Fundamentals of Natural Gas Processing.

[5] Bernardo P., Drioli E., Golemme G. (2009). Membrane Gas Separation: A Review/State of the Art. Ind. Eng. Chem. Res..

[6] Scholes C.A., Stevens G., Kentish S. (2012). Membrane gas separation applications in natural gas processing. Fuel.

[7] Seader J.D., Henley E.J., Roper D.K. (2005). Separation Process Principles.

[8] Dortmundt D., Doshi K. (2003). Recent Developments in CO_2_ Removal Membrane Technology.

[9] Baker R.W. (2002). Future Directions of Membrane Gas Separation Technology. Ind. Eng. Chem. Res..

[10] Davis R.A. (2002). Simple Gas Permeation and Pervaporation Membrane Unit Operation Models for Process Simulators. Chem. Eng. Technol..

[11] Rautenbach R., Knauf R., Struck A., Vier J. (1996). Simulation and design of membrane plants with AspenPlus. Chem. Eng. Technol..

[12] Rautenbach R., Albrecht R. (1989). Membrane Processes.

[13] Pan C.Y. (1983). Gas separation by permeators with high-flux asymmetric membranes. AIChE J..

[14] Qi R., Henson M.A. (1996). Approximate modeling of spiral-wound gas permeators. J. Membr. Sci..

[15] Krovvidi K.R., Kovvali A.S., Vemury S., Khan A.A. (1992). Approximate solutions for gas permeators separating binary mixtures. J. Membr. Sci..

[16] Safari M., Ghanizadeh A., Montazer-Rahmati M.M. (2009). Optimization of membrane-based CO_2_-removal from natural gas using simple models considering both pressure and temperature effects. Int. J. Greenh. Gas Control..

[17] Lin D., Ding Z., Liu L., Ma R. (2012). Modeling spiral-wound membrane modules with applications for gas/vapor permeation. Comput. Chem. Eng..

[18] Gholami G., Soleimani M., Ravanchi M.T. (2015). Mathematical Modeling of Gas Separation Process with Flat Carbon Membrane. J. Membr. Sci. Res..

[19] Qadir S., Hussain A., Ahsan M. (2019). A Computational Fluid Dynamics Approach for the Modeling of Gas Separation in Membrane Modules. Processes.

[20] Dias A.C.S., Sá M.C.C.D., Fontoura T.B., Menezes D.Q., Anzai T.K., Diehl F.C., Thompson P.H., Pinto J.C. (2020). Modeling of spiral wound membranes for gas separations. Part I: An iterative 2D permeation model. J. Membr. Sci..

[21] Thundyil M.J., Koros W.J. (1997). Mathematical modeling of gas separation permeators—for radial crossflow, countercurrent, and cocurrent hollow fiber membrane modules. J. Membr. Sci..

[22] Marriott J., Sørensen E. (2003). A general approach to modelling membrane modules. Chem. Eng. Sci..

[23] Qi R., Henson M.A. (1997). Modeling of Spiral-Wound Permeators for Multicomponent Gas Separations. Ind. Eng. Chem. Res..

[24] Pan C.Y. (1986). Gas separation by high-flux, asymmetric hollow-fiber membrane. AIChE J..

[25] Boudinar M., Hanbury W., Avlonitis S. (1992). Numerical simulation and optimisation of spiral-wound modules. Desalination.

[26] van der Meer W., van Dijk J. (1997). Theoretical optimization of spiral-wound and capillary nanofiltration modules. Desalination.

[27] Ahmad F., Lau K.K., Shariff A.M. (2010). Modeling and Parametric Study for CO_2_/CH_4_ Separation using Membrane Processes. World Acad. Sci. Eng. Technol..

[28] Davidson T.A. (1993). A Simple and Accurate Method for Calculating Viscosity of Gaseous Mixtures.

[29] Pandey J., Mukherjee S., Yadav M.K., Dey R. (2005). Viscosity of multicomponent gas mixtures. J. Indian Chem. Soc..

[30] Ghosh T., Prasad D., Dutt N., Rani K.Y. (2007). Viscosity of Liquids: Theory, Estimation, Experiment, and Data.

[31] Reid R.C., Prausnitz J.M., Poling B.E. (1987). The Properties of Gases and Liquids.

[32] Baker R.W., Bell C.M., Chow P., Louie J., Mohr J.M., Peinemann K.V., Pinnau I., Wijmans J.G., Gottschlich D.E., Roberts D.L. (1990). Low Cost Hydrogen/Novel Membrane Technology for Hydrogen Separation from Synthesis Gas.

[33] Tan L.S., Lau K.K., Bustam M.A., Shariff A.M. (2012). Removal of high concentration CO_2_ from natural gas at elevated pressure via absorption process in packed column. J. Nat. Gas Chem..

[34] Alqaheem Y., Alomair A., Vinoba M., Pérez A. (2017). Polymeric Gas-Separation Membranes for Petroleum Refining. Int. J. Polym. Sci..

[35] Lee A., Feldkirchner H., Stern S., Houde A., Gamez J., Meyer H. (1995). Field tests of membrane modules for the separation of carbon dioxide from low-quality natural gas. Gas Sep. Purif..

[36] Qi R., Henson M. (1998). Optimization-based design of spiral-wound membrane systems for CO_2_/CH_4_ separations. Sep. Purif. Technol..

[37] Merkel T., Amo K., Baker R., Daniels R., Friat B., He Z., Lin H., Serbanescu A. (2009). Membrane Process to Sequester CO_2_ from Power Plant Flue Gas.

[38] Karode S.K., Kumar A. (2001). Flow visualization through spacer filled channels by computational fluid dynamics I.: Pressure drop and shear rate calculations for flat sheet geometry. J. Membr. Sci..

[39] Schock G., Miquel A. (1987). Mass transfer and pressure loss in spiral wound modules. Desalination.

[40] Johnson J.E. (2013). Design and Construction of Commercial Spiral Wound Modules. Encyclopedia of Membrane Science and Technology.

[41] Hussain A., Hägg M.-B. (2010). A feasibility study of CO_2_ capture from flue gas by a facilitated transport membrane. J. Membr. Sci..

[42] White L.S., Wei X., Pande S., Wu T., Merkel T.C. (2015). Extended flue gas trials with a membrane-based pilot plant at a one-ton-per-day carbon capture rate. J. Membr. Sci..

[43] Salim W., Vakharia V., Chen Y., Wu D., Han Y., Ho W.W. (2018). Fabrication and field testing of spiral-wound membrane modules for CO2 capture from flue gas. J. Membr. Sci..

[44] Han Y., Salim W., Chen K.K., Wu D., Ho W.W. (2019). Field trial of spiral-wound facilitated transport membrane module for CO_2_ capture from flue gas. J. Membr. Sci..

